# Quantifying the impact of symptomatic acute hepatic porphyria on well‐being via patient‐reported outcomes: Results from the Porphyria Worldwide Patient Experience Research (POWER) study

**DOI:** 10.1002/jmd2.12343

**Published:** 2022-10-18

**Authors:** Amy Dickey, Kristen Wheeden, Desiree Lyon, Sue Burrell, Sean Hegarty, Rocco Falchetto, Edrin R. Williams, Jasmin Barman‐Aksözen, Marc DeCongelio, Alison Bulkley, Joana E. Matos, Tarek Mnif, Jordanna Mora, John J. Ko, Stephen Meninger, Stephen Lombardelli, Danielle Nance

**Affiliations:** ^1^ Division of Pulmonary and Critical Care Medicine, Department of Medicine Massachusetts General Hospital Boston Massachusetts USA; ^2^ American Porphyria Foundation Bethesda Maryland USA; ^3^ Global Porphyria Advocacy Coalition Durham City UK; ^4^ Swiss Society for Porphyria Zürich Switzerland; ^5^ Institute of Laboratory Medicine, Department of Medical Institutes Stadtspital Zürich, Triemli Zürich Switzerland; ^6^ Cerner Enviza (formerly Kantar Health) New York New York USA; ^7^ Cerner Enviza (formerly Kantar Health) Kansas City Missouri USA; ^8^ Cerner Enviza (formerly Kantar Health) Paris France; ^9^ Alnylam Pharmaceuticals Cambridge Massachusetts USA; ^10^ Alnylam Pharmaceuticals Maidenhead UK; ^11^ Banner Health Gilbert Arizona USA

**Keywords:** acute hepatic porphyria, acute intermittent porphyria, acute porphyria, AHP, patient‐reported outcomes, survey

## Abstract

Acute hepatic porphyria (AHP) is a group of rare genetic diseases of heme biosynthesis resulting in severe neurovisceral attacks and chronic complications that negatively impact patients' well‐being. This study evaluated the impacts of AHP on patients' physical and emotional health from a global perspective. Adult patients from the United States, Italy, Spain, Australia, Mexico, and Brazil with AHP with >1 porphyria attack within the past 2 years or receiving intravenous hemin and/or glucose for attack prevention completed an online survey assessing demographics, health characteristics, and patient‐reported outcomes. Results were analyzed collectively and by patient subgroups. Ninety‐two patients with AHP across the six countries completed the survey. More than 70% of patients reported that their physical, emotional, and financial health was fair or poor. Among patients who reported pain, fatigue, and muscle weakness, 94.3%, 95.6%, and 91.4%, respectively, reported that these symptoms limited daily activities. Moderate to severe depression was present in 58.7% of patients, and moderate to severe anxiety in 48.9% of patients. Of the 47% of patients who were employed, 36.8% reported loss in productivity while at work. Among patients, 85.9% reported that they had to change or modify goals that were important to them because of AHP. Aside from differences in healthcare utilization and pain severity, scores did not significantly vary with attack rate or use of hemin or glucose prophylactic treatments. AHP substantially impacts patients' physical and emotional well‐being, regardless of hemin or glucose prophylactic treatment or frequency of attacks. This multinational study demonstrates that there is substantial disease burden for patients with AHP, even among those experiencing sporadic attacks or using prophylactic treatment.

**Synopsis**

This multinational study demonstrates that there is substantial disease burden for patients with acute hepatic porphyria (AHP), even among those experiencing sporadic attacks or using prophylactic treatment.

## 1 INTRODUCTION

Acute hepatic porphyrias (AHP) are a group of rare genetic diseases of heme biosynthesis.^1^, ^2^ Four types of acute porphyria have been identified, each having unique enzymatic defects: acute intermittent porphyria (AIP), hereditary coproporphyria (HCP), variegate porphyria (VP), and ALAD (5′‐aminolevulinic acid dehydratase) deficiency porphyria (ADP).^1^ Among these AHP types, AIP is the most common, with a calculated prevalence of 5.9 per 1 million in European countries.^3^ Females of reproductive age are most commonly affected.^4^

AHP is characterized by potentially life‐threatening attacks that often include pain, most frequently abdominal. These attacks may be provoked by triggering factors such as medications, hormones, or stress.^4^ Patients may also present with seizures, psychological symptoms, chronic pain, and neuropathy.^5^ Associations have been found between AHP and long‐term complications (e.g., hypertension, chronic kidney disease, hepatocellular carcinoma).^6^

A majority of patients with pathogenic AHP variants have latent disease and do not experience attacks, but others have attacks that may occur sporadically or more frequently despite avoidance of triggering factors.^7^ Chronic symptoms may occur in patients with both recurrent and sporadic attacks; however, previous studies suggest that the prevalence of chronic symptoms is higher in those patients experiencing more frequent attacks.^7^, ^8^, ^9^ Symptom prevention strategies include trigger avoidance, suppression of ovulation, and off‐label hemin prophylaxis.^9^ Treatment options for acute attacks include carbohydrate loading with glucose and hemin.^10^, ^11^, ^12^ Givosiran is a subcutaneously administered RNA interference therapeutic approved for AHP treatment in adults in the United States, Brazil, and Canada^13^, ^14^, ^15^ and in adults and adolescents age ≥ 12 years in the European Union, Switzerland, and Japan.^16^, ^17^, ^18^

Much of the literature on AHP is focused on acute attacks and clinical manifestations. The prospective, observational EXPLORE study, conducted in the United States and Europe, was designed to characterize the clinical management of AHP patients who experience recurrent attacks.^9^ Two thirds of patients reported chronic symptoms between attacks, with nearly half reporting daily symptoms regardless of whether they were receiving off‐label hemin prophylaxis for prevention of attacks.

AHP is associated with poor quality of life (QoL).^19^ In patient interviews, 16 US patients described symptoms of generalized pain, confusion, irritability, and fatigue. Patients also indicated that their condition increased their feelings of isolation and had a negative effect on their relationships with other people.^19^, ^20^ Another study of 19 US patients found that the disease manifestations had an impact on patients' day‐to‐day living, including worsening sleep, increasing use of the healthcare system, and limiting social interactions and the ability to function at work.^20^

For patients, caregivers, and clinicians, these studies and others provide insight into the extent of the impact of AHP beyond clinical symptoms.^21^ While prior studies evaluated a limited set of patient‐reported outcome (PRO) tools among patients within particular countries,^2^, ^22^, ^23^, ^24^ there is still a lack of understanding of the full burden of AHP, especially in a study including a diverse set of patients across the globe. The present study was designed to assess the full spectrum of the AHP patient's well‐being and unmet emotional, mental, and physical needs across countries and regions of the world.

## 2 PATIENTS AND METHODS

### 2.1 Study design

For this multinational survey, adults with AHP were recruited via patient advocacy groups (PAGs) and physicians in the United States, Italy, Spain, Australia, Mexico, and Brazil. The PAGs and physicians used email and social media to distribute a unique survey link to patients. Before beginning the survey, all patients provided informed consent electronically. Study materials were reviewed by Sterling Institutional Review Board, which determined the study met exemption status.

Patients were included in the study if they met the following criteria: age ≥ 18 years, self‐reported AHP diagnosis, diagnostic test result confirming diagnosis, willingness to provide medical records to confirm diagnosis (though medical records were not actually obtained), ≥1 AHP attack within past 2 years or taking routine or scheduled hemin/heme arginate (intravenous [IV] hemin therapy), taking on‐demand hemin/heme arginate (as needed for an attack), taking routine or scheduled IV glucose, or taking on‐demand IV glucose (as needed for an attack). Treatments evaluated were not mutually exclusive—patients could be receiving more than one. Patients who were taking givosiran or who were unable or unwilling to provide informed consent were excluded.

Each participant completed the online survey. The questionnaire, administered in the primary language of each country and completed via a secure encrypted system, took an average of 30 minutes to complete. Data analysis was conducted only for participants who completed the entire survey.

### 2.2 Study questionnaire

The survey was designed by patients, physicians, PAGs, and researchers using standardized surveys, as well as de novo questions. Before the survey design was finalized, pretest cognitive debriefing interviews were conducted with three patients to obtain feedback on survey content and to ensure quality data collection.

Sociodemographic characteristics collected included country of residence, gender, employment status, marital status, family and living situation, occurrence of menstrual cycles, and impact of COVID‐19 (Supplementary Table 1).

Health‐related variables included AHP date of diagnosis, AHP diagnostic tests, AHP treatments, age at first symptoms, age at first diagnosis, time to diagnosis, age at first treatment, duration of active disease, time since first treatment, time receiving IV hemin therapy, time receiving IV glucose therapy, number of AHP attacks within the past 2 years, and number of attacks leading to hospitalization, an emergency department visit, or a doctor visit within the past 2 years (Supplementary Table 2).

Patient well‐being was evaluated by assessing the personal impact of AHP symptoms via PROs. Supplementary Table 3 lists the categorical PRO variables collected. Acute and chronic symptoms were ranked by level of burden on daily activities. Chronic symptom severity and health perceptions were measured on a Likert scale. Continuous PRO variables are listed in Supplementary Table 4. The 8‐item Patient Health Questionnaire depression scale (PHQ‐8; scale, 0–24) and the 7‐item Generalized Anxiety Disorder scale (GAD‐7; scale, 0–21) were used to screen patients for depression and anxiety, respectively. Current moderate to severe depression was identified with a PHQ‐8 cutoff score of ≥10,^25^ and mild, moderate, and severe anxiety was identified with GAD‐7 scores of 5, 10, and 15, respectively.^26^

The West Haven–Yale Multidimensional Pain Inventory (WHYMPI) was used to examine the impact of pain on patients' lives, with responses indicated on a 7‐point Likert scale. The Work Productivity and Activity Impairment (WPAI) instrument was used to evaluate absenteeism (defined as employee's absence from work because of their health), presenteeism (defined as employee's being physically present at work but not fully functional because of their health), overall work productivity impairment, and activity impairment. Only respondents who reported being employed completed the absenteeism, presenteeism, and overall work impairment items. All respondents completed the activity impairment item. Additional de novo questions regarding the patients' physical, emotional, and social health were also evaluated (Supplementary Table 5).

### 2.3 Data analysis

Patient demographic and health history information was reported descriptively using counts, means, and standard deviations for continuous variables and frequencies and percentages for categorical variables. Bivariate analysis was performed to evaluate differences between patient subgroups.

Subgroup analyses were performed between patients with sporadic versus recurrent attacks. Sporadic attacks were defined as 0–5 attacks within 2 years, and recurrent attacks as ≥6 attacks within 2 years.

Patients receiving prophylactic treatment were compared to those not receiving prophylactic treatment. The prophylactic treatment subgroup included all patients on routine/scheduled hemin, routine/scheduled IV glucose, and/or gonadotropin‐releasing hormone agonist. The non‐prophylactic subgroup included all patients not receiving any of these treatments. Treatment selections were not mutually exclusive.

Independent *t* tests were used to compare continuous variables between subgroups, and *χ* ^2^ tests were used to compare categorical variables. Sample size requirements for bivariate comparisons were estimated assuming 80% power and an α value of 0.05. Based on empirical differences in means in prior studies, sample size requirements for detecting statistically significant differences in subgroup comparisons were *N* = 86 for mental health and *N* = 38 for physical functioning.^22^ Statistical analysis was performed with SAS and SPSS statistics software.

## 3 RESULTS

### 3.1 Patient characteristics

In total, 2375 individuals with AHP or caregivers were sent the study link by PAGs and physicians to participate. Of 328 AHP patients who provided informed consent, 92 (28%) accessed the screener, met the inclusion criteria, and completed the survey. The patients with AHP who completed the survey were residents of Brazil (29.3%), the United States (21.7%), Spain (17.4%), Italy (12.0%), Australia (9.8%), and Mexico (9.8%). The mean (SD) age was 41.1 (12.4) years, 90.2% were female, and a third (34.7%) of the participants reported being unemployed or disabled. While the mean age at AHP diagnosis was 30.8 years, the mean time to diagnosis was 6.4 years. Most patients (73.9%) had a diagnosis of AIP (Table 1). Other sociodemographic characteristics data are listed in Supplementary Table 1.

**TABLE 1:** Patient demographics and health characteristics

Characteristic	Total sample (*N* = 92)
Age, mean (SD), years	41.1 (12.4)
Female, n (%)	83 (90.2)
Diagnosis, n (%)	
Acute intermittent porphyria	68 (73.9)
Hereditary coproporphyria	12 (13.0)
Variegate porphyria	9 (9.8)
5′‐Aminolevulinic acid dehydratase deficiency porphyria	1 (1.1)
Age at diagnosis, mean (SD), years	30.8 (10.8)
Age at first symptoms, mean (SD), years	24.3 (10.7)
Time to diagnosis, mean (SD), years	6.4 (10.1)
Duration of disease, mean (SD), years	16.9 (13.0)
AHP attacks within past 2 years, median (IQR)	4.5 (2, 12)
AHP attacks leading to hospitalization, median (IQR)	2.0 (0, 3)
AHP treatment, n (%)	
Trigger avoidance	59 (64.1)
On‐demand IV glucose, as needed for an attack	52 (56.5)
On‐demand hemin, as needed for an attack	36 (39.1)
Routine/scheduled hemin	22 (23.9)
Routine/scheduled IV glucose	18 (19.6)
Holistic therapies	16 (17.4)
Gonadotropin‐releasing hormone agonist	2 (2.2)

Abbreviations: AHP, acute hepatic porphyria; IQR, interquartile range; IV, intravenous; SD, standard deviation.

Patients reported a variety of treatments and management strategies, including trigger avoidance (64.1%), on‐demand IV glucose (56.5%), and on‐demand hemin (39.1%) (Table 1). Prophylactic treatment, including hemin and/or glucose, was used by 38% of patients. The mean age at first symptoms was 24.3 years, and the mean (SD) duration of active disease to date was 16.9 (13.0) years (Table 1). Patients experienced a median of 4.5 AHP attacks within the 2 years before survey participation (Table 1), and a median of 2 of these attacks led to hospitalization or emergency department visits.

### 3.2 Current health perception

**FIGURE 1:**
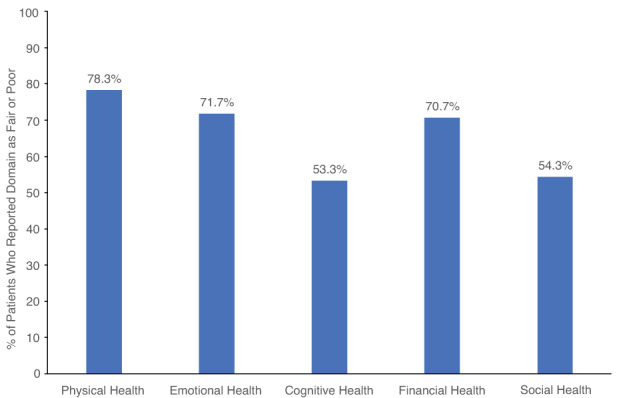
Current health perception. Each health aspect was categorized as poor, fair, good, very good, or excellent. Graph displays percentages of patients who reported fair or poor perception of their health for each domain

Among physical, emotional, cognitive, financial, and social health domains, AHP patients reported the greatest impairment on physical, emotional, and financial health, with 70% or more of patients reporting their physical, emotional, or financial health as fair or poor (Figure 1).

### 3.3 Physical health

**FIGURE 2:**
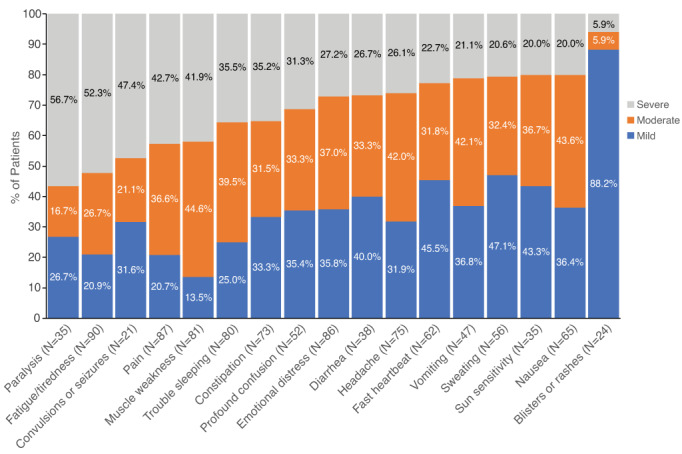
Patients reporting mild, moderate, or severe impact of symptoms on daily activities. Results recorded among patients who experienced symptoms

When asked to identify their most burdensome acute symptoms, 71.7% of patients reported acute pain, 37.0% reported acute muscle weakness, and 28.3% reported acute fatigue. The same symptoms were reported as the most burdensome chronic symptoms. A moderate to severe impact on daily activities was reported among 79.3% of patients who experienced chronic pain, 86.5% who experienced chronic muscle weakness, and 79.0% who experienced chronic fatigue. Trouble sleeping also interfered with 87% of patients' daily life, with 75.0% of these reporting a moderate to severe impact (Figure 2). On the WHYMPI, which measures important dimensions of the chronic pain experience (scale, 0–6, lowest to highest impact), patients with AHP reported a mean score of 3.6 for interference of pain in vocational, social/recreational, and family/marital functioning, 4.5 for perceptions of support received by others, 3.4 for pain severity, 3.3 for perceived life control, and 3.7 for level of affective distress (Supplementary Table 6). Additionally, 37.0% of patients in the study agreed or strongly agreed that their pain no longer responds well to therapy.

### 3.4 Social, emotional, and mental health

**FIGURE 3:**
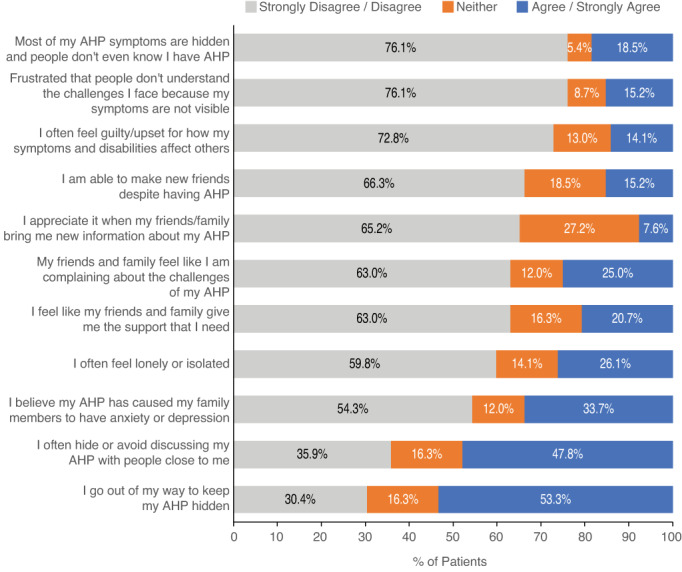
Impact of AHP on social life. Participants were asked a series of questions regarding the impact of AHP on their social life. Percentages of patients with responses of strongly disagree/disagree, neither, and agree/strongly agree are displayed. AHP, acute hepatic porphyria

**FIGURE 4:**
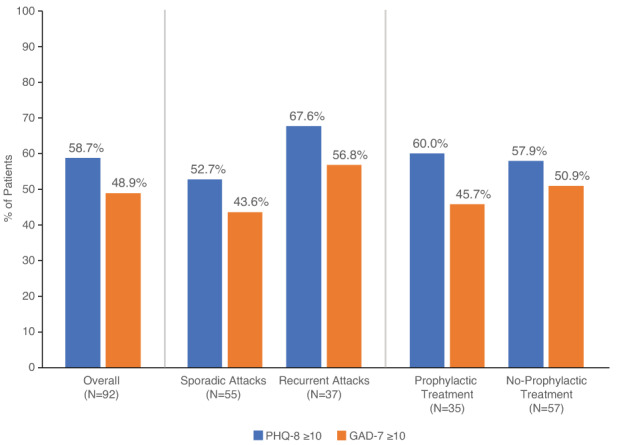
Percentages of AHP patients with moderate to severe depression and anxiety. PHQ‐8 and GAD‐7 scores ≥10 are displayed collectively and by both attack rate and use of prophylactic treatment. No statistical difference was detected between groups. GAD‐7, 7‐item Generalized Anxiety Disorder scale (range, 0–21); PHQ‐8, 8‐item Patient Health Questionnaire depression scale (range, 0–24)

Regarding social life, a majority of patients (59.8%) reported often feeling lonely or isolated, and many patients (72.8%) often felt guilty and upset about how their symptoms and disabilities affected those around them. However, a majority of patients (63.0%) also felt their friends and family gave them the support they need (Figure 3). When screened for depression and anxiety, patients had a mean (SD) score of 12.1 (6.7) on the PHQ‐8 (scale range, 0–24) and a mean (SD) score of 10.3 (5.7) on the GAD‐7 (scale range, 0–21). On the PHQ‐8, 54 (58.7%) patients scored at least 10, indicating moderate to severe depression (Figure 4). On the GAD‐7, 45 (48.9%) patients scored at least 10, indicating moderate to severe anxiety. Mild anxiety (GAD‐7: 5–9) was reported by 33.7% of patients, moderate anxiety (GAD‐7: 10–14) by 21.7% of patients, and severe anxiety (GAD‐7: 15+) by 27.2% of patients. Regarding mental acuity, 70.7% of patients agreed or strongly agreed that they sometimes forget names or words for common objects, and 63.1% agreed or strongly agreed that they sometimes have trouble making decisions (Supplementary Figure 1). Regarding participants' personal life/goals, most participants (85.9%) agreed or strongly agreed that they had to change or modify goals that were important to them because of AHP. Many patients (71.8%) also reported that they are committed to a healthy diet and exercise to prevent AHP symptoms. Approximately half (55.5%) of patients agreed or strongly agreed that AHP pushed them to find a new sense of purpose (Supplementary Figure 2).

### 3.5 Financial health

On the WPAI, which was used to assess work productivity and overall activity impairment (expressed as impairment percentages with higher numbers indicating greater impairment), employed participants (N = 43) reported missing 32.6% of work time in the previous week as well as a 36.8% loss in productivity while at work because of their AHP (Supplementary Table 7). Overall work productivity impairment was 52.3% among employed participants, and the overall activity impairment score was 51.6% among all patients. Additionally, 60.9% of participants agreed or strongly agreed that AHP affected their ability to keep their job. Similarly, 72.9% reported that they agreed or strongly agreed with the feeling of not being able to work to their full potential because of AHP.

### 3.6 Sporadic attack versus recurrent attack subgroups

Of the 92 AHP patients who completed the survey, 55 (60%; mean age, 40.3 years) reported sporadic attacks and 37 (40%; mean age, 42.3 years; Supplementary Table 8) reported recurrent attacks. Among the sporadic attack group, 92.7% were female; among the recurrent attack group, 86.5%. Among the sporadic and recurrent attack groups, respectively, 85.2% and 61.1% had a diagnosis of AIP. Physical, emotional, cognitive, financial, and social health were perceived negatively by both subgroups, with no difference between groups (Supplementary Figure 3). A majority of patients in the sporadic and recurrent attack groups reported a PHQ‐8 score ≥ 10, indicating moderate to severe depression (52.7% and 67.6%, respectively; Figure 4). Among patients in the sporadic and recurrent groups, 43.6% and 56.8%, respectively, reported a GAD‐7 score ≥ 10, and 25.5% and 29.7%, respectively, reported a GAD‐7 score ≥ 15, indicating severe anxiety. Pain was reported as one of the top three most burdensome chronic symptoms in the sporadic (50.9%) and recurrent (59.5%) groups. Work productivity was similar across attack rates (Supplementary Table 7).

While no significant differences were present in other domains, patients in the sporadic and recurrent attack subgroups differed in healthcare utilization and pain severity (Supplementary Table 9). The mean (SD) number of attacks leading to hospitalization or to emergency department visits in patients with sporadic attacks was 1.63 (1.45) and 1.61 (1.51), compared to 4.00 (4.50) and 6.41 (7.60), respectively, in patients with recurrent attacks (*P* < 0.001 for both). The mean (SD) WHYMPI score (scale, 0–6) in patients with sporadic attacks was 2.93 (1.68), compared to 4.17 (1.25) in patients with recurrent attacks (*P* < 0.001; Supplementary Table 6).

### 3.7 Prophylactic treatment versus no prophylactic treatment subgroups

Thirty‐five patients (38%; mean age, 41.0 years) reported use of prophylactic treatment with glucose or hemin, and 57 (62%; mean age, 41.1 years) reported no prophylactic treatment. Among the prophylactic group, 88.6% were female; among the no‐prophylactic group, 91.2%. Among the prophylactic and no‐prophylactic groups, respectively, 58.8% and 85.7% had a diagnosis of AIP. Health perceptions among patients in the prophylactic/no‐prophylactic treatment subgroups are shown in Supplementary Figure 4. A majority of patients in both the prophylactic and no‐prophylactic groups reported a PHQ‐8 score ≥ 10, indicating moderate to severe depression (60.0% and 57.9%, respectively; Figure 4). Among patients in the prophylactic and no‐prophylactic groups, 45.7% and 50.9%, respectively, reported a GAD‐7 score ≥ 10, signifying moderate to severe anxiety. Pain was reported as one of the top three most burdensome chronic symptoms in the prophylactic (51.4%) and no‐prophylactic (56.1%) groups. While there were no significant differences in other domains, AHP patients who received prophylactic treatment experienced three times more emergency department visits because of attacks compared with those who did not receive prophylaxis for AHP (1.83 [SD = 2.10] for no vs. 6.78 [SD = 7.93] for yes; *P* < 0.001; Supplementary Table 9).

## 4 DISCUSSION

This survey study was designed to broadly characterize the psychosocial well‐being of a large number of AHP patients around the world. With use of standardized and de novo questions, QoL was evaluated across a wide range of domains, including patient's current health perceptions, burdensomeness of symptoms, symptom impact on daily activities, impact and severity of pain, social functioning, anxiety, depression, cognitive functioning, work performance, life goals, and healthcare utilization. The results demonstrated a remarkable impact of AHP across all domains that did not vary with attack rate or use of hemin or glucose prophylaxis.

Notably, a large proportion of patients reported negative perceptions regarding their physical, emotional, and financial health. The most burdensome symptoms identified—pain, muscle weakness, and fatigue—are similar to those reported in prior survey/interview studies.^19^, ^20^ The present study found that these symptoms had a moderate to severe impact on daily activities.

Results from the present study suggest that, compared with the general population, patients living with AHP experience substantially more symptoms of depression and anxiety. When evaluated with standardized depression and anxiety scales, the AHP patients in this study were found to experience significant rates of moderate to severe depression and anxiety (58.7% and 48.9%, respectively). Among the subgroups examined, rates of moderate to severe depression and anxiety ranged from 43.6% to 67.6%, suggesting a substantial mental health burden regardless of attack rates or AHP prophylaxis. These rates are substantially higher than those in a 2019 US national survey in which 7% of adults reported moderate or severe depression (PHQ‐8 score ≥ 10) within the preceding 2 weeks, and 9.5%, 3.4%, and 2.7% reported mild, moderate, and severe anxiety symptoms (GAD‐7 scores 5–9, 10–14, and 15–21), respectively.^27^, ^28^

Findings from this study suggest that the impact of the pain experienced by patients with AHP is considerable. In fact, pain was reported to be the most burdensome acute and chronic symptom they experience. Pain limited the daily activities of 94.3% of respondents, and 37% of patients in the study reported that their pain no longer responds well to therapy. The pain reported in the present study is comparable to that experienced by patients with other causes of chronic pain. In one study, 120 patients with chronic pain not associated with AHP reported mean WHYMPI scores (scale, 0–6) of 3.74, 4.31, 3.55, 3.63, and 3.23 on subscales of interference, support, pain severity, self‐control, and affective distress, respectively.^29^ Patients were heterogeneous with regard to etiology and site of the primary pain complaint, although 36.4% complained of low back pain. On average, patients had experienced constant pain for 10.2 years, 55.8% had had at least one pain‐related surgery, and 67.4% were taking pain medication.^29^

In the EXPLORE study, 68% of recurrent‐attack patients reported being unable to work full‐time because of AHP.^9^ Of employed patients, 52% reported missing many workdays as a result of AHP (average 40.2 workdays lost within past year).^9^ Similarly, the current study results showed significant unemployment rates and work productivity impairment regardless of attack rate or AHP treatment. Overall activity impairment was 51.6% among all patients; 60.9% of participants thought AHP affected their ability to keep a job; and 72.9% thought they could not work to their full potential because of AHP.

Although all patients surveyed had an AHP diagnosis, not all were receiving active prophylactic treatment for their symptoms. Many patients relied only on trigger avoidance and on‐demand treatment, which is especially notable given that patients experienced a median of 4.5 attacks within the past 2 years. The subgroup analyses comparing sporadic and recurrent attack groups found they were similar in terms of perceived physical, emotional, cognitive, financial, and social health; depression and anxiety; and absenteeism and presenteeism. The groups differed in healthcare utilization and pain severity, with the recurrent attack group experiencing more healthcare utilization and severe pain. Similar patterns were observed between the prophylactic and no‐prophylactic subgroups, with those on hemin or glucose prophylaxis experiencing more healthcare utilization. Neither hemin nor glucose has been approved for AHP symptom prophylaxis, and the data indicate they are insufficient for prophylaxis in AHP.

The mean time to diagnosis was 6.4 years.^30^ Possible factors contributing to this delayed diagnosis are the nonspecific symptoms of AHP, the limited disease awareness, and the transience of severe symptoms. Patients with undiagnosed AHP worsen while waiting and undergoing treatments for an incorrect diagnosis. Untreated attacks can lead to severe neurologic damage, long recovery, and fatality.^9^ Therefore, an earlier diagnosis of AHP would be expected to reduce lifetime disease burden, decrease the negative psychosocial impact, improve patient well‐being, and limit unnecessary treatments and hospitalizations.

This survey study is unique in that it was developed with the AHP community, including patients, physicians, and PAGs, which ensured relevance and meaningfulness of results collected. Moreover, data were collected from a multinational real‐world patient sample, resulting in high external validity of the study results. The breadth of this survey provided additional insight into the overall patient burden of AHP, adding evidence to the growing body of literature on the clinical burden of AHP. This may aid physicians and caregivers in factoring in patient's particular QoL challenges and mental health needs when considering care for this patient population. Additionally, these results may inform the development of educational and support programming for patients with AHP and their families.

Despite its strengths, this study also has some limitations. First, the cross‐sectional nature of the study limits the ability to demonstrate causal relationships. Second, PROs are limited by the subjective nature of reporting. Third, the diagnosis of AHP was reported by the participants and not confirmed by a physician. Fourth, the study presents survey data and thus may be subject to response bias. Fifth, the study presents data from patients who completed the entire survey—thus introducing ascertainment bias, as patients who did not complete the full 30‐minute survey are not represented in the final data analysis. Sixth, because the survey excluded patients taking givosiran and because patients with recurrent attacks are more likely to be taking givosiran, the study may have selected patients with a milder disease phenotype. Seventh, the comparison between patients on or off prophylaxis with hemin or glucose was not able to control for the baseline severity of disease in these groups without prophylaxis. Last, the COVID‐19 pandemic may have influenced study results in that 23.9% of patients reported COVID‐19 had some impact on their condition and/or treatment.

## 5 CONCLUSION

These multinational results demonstrate that AHP disease burden, even among those experiencing sporadic attacks or using prophylactic hemin/glucose, is substantial. Similarly, the present results indicate that the disease burden not only affects patients' physical health, but also their emotional, social, and financial well‐being. These findings highlight the need for therapeutic strategies that can reduce attacks, alleviate chronic disease manifestations, and improve patient well‐being over time.

## FUNDING INFORMATION

This work was supported by Alnylam Pharmaceuticals, Cambridge, MA, USA. Data collection and analysis were performed by Kantar Health (now Cerner Enviza) under the direction of the authors. Funding for this study was provided by the sponsor, Alnylam Pharmaceuticals. The authors developed the protocol, interpreted the data, collaborated in manuscript preparation, and approved the submission of the article for publication. Medical writing and editorial assistance were provided by Peloton Advantage, LLC, an OPEN Health company, in accordance with Good Publication Practice (GPP3) guidelines and funded by Alnylam Pharmaceuticals. The authors confirm independence from the sponsors; the content of the article has not been influenced by the sponsors.

## CONFLICT OF INTEREST

The authors declare the following competing interests: Amy Dickey had speaking engagements and received consulting honoraria from Alnylam Pharmaceuticals for participation in this research and for other porphyria‐related consulting. Kristen Wheeden was employed by the American Porphyria Foundation at the time of the study and currently is president of the United Porphyrias Association; she received grant and sponsorship funding to the American Porphyria Foundation from Alnylam Pharmaceuticals and served on a medical advisory board for Alnylam Pharmaceuticals. Desiree Lyon received grant and sponsorship funding to the American Porphyria Foundation from Alnylam Pharmaceuticals. Sue Burrell received grant and sponsorship funding to the British Porphyria Association as well as consulting honoraria from Alnylam Pharmaceuticals for participation on various patient advisory group leadership advisory boards. Sean Hegarty, Rocco Falchetto, and Jasmin Barman‐Aksözen report no competing interests. Edrin R. Williams was employed by the American Porphyria Foundation at the time of the study. Marc DeCongelio, Alison Bulkley, Joana E. Matos, and Tarek Mnif were employed at the time of the study by Kantar Health (now Cerner Enviza), a company that provides consultancy support to Alnylam. Jordanna Mora and John J. Ko were employed by Alnylam Pharmaceuticals at the time of the study and own stock and stock options in the company; currently they are employed by Beam Therapeutics and own stock and stock options in the company. Stephen Meninger and Stephen Lombardelli are employed by and own stock and stock options in Alnylam Pharmaceuticals. Danielle Nance served on advisory boards for Aptevo Therapeutics, Bayer, HemaBiologics, and Medexus Pharmaceuticals; served on speaker bureaus for BPL and the National Hemophilia Foundation; provided consulting services to Goval; served as an author for Bayer; and had speaking engagements and received consulting honoraria from Alnylam Pharmaceuticals for participation in this research.

## ETHICS STATEMENT

Study materials were reviewed by Sterling Institutional Review Board, which determined the study met exemption status.

## PATIENT CONSENT

Prior to participation, all patients provided informed consent electronically.

## ANIMAL RIGHTS

Not applicable.

## Supporting information


**Appendix S1.** Supporting information

## Data Availability

De‐identified individual participant data that support these results will be made available in a secure‐access environment 12 months after study completion. Access will be provided contingent upon the approval of a research proposal and the execution of a data sharing agreement.
