# CORRIGENDUM

**DOI:** 10.1002/jmd2.12376

**Published:** 2023-07-02

In the following article,^1^ there were errors in Figure 3 and Supplementary Figure 2. In both figures, the “Strongly Disagree / Disagree” and “Agree / Strongly Agree” results were transposed. Both figures have been corrected.

**FIGURE 3:**
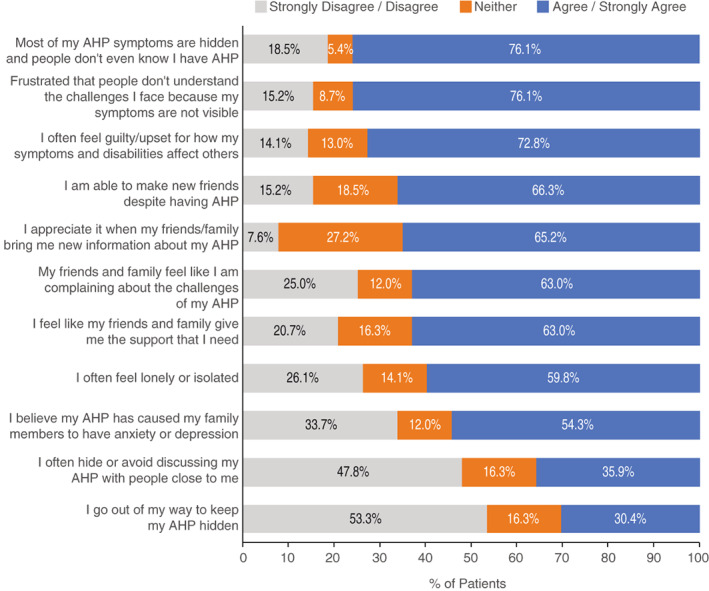


The correct Figure 3 is shown below:

The Supplementary Figure 2 has been corrected online.

The authors apologize for the errors.
