# Supplementary material for: Quantifying the impact of symptomatic acute hepatic porphyria on well‐being via patient‐reported outcomes: Results from the Porphyria Worldwide Patient Experience Research (POWER) study
Source: JIMD Rep. 2022 Oct 18;64(1):104–13. doi: 10.1002/jmd2.12343 (PMC9830021; doi:10.1002/jmd2.12343)
Supplement: Supplementary file 1 — Appendix S1. Supporting information [file JMD2-64-104-s001.docx]

**SUPPLEMENTARY MATERIAL FOR**

Quantifying the Impact of Symptomatic Acute Hepatic Porphyria on Well-Being via
Patient-Reported Outcomes: Results from the Porphyria Worldwide
Patient Experience Research (POWER) Study

Amy Dickey^1^, Kristen Wheeden^2^, Desiree Lyon^2^, Sue Burrell^3^, Sean Hegarty^3^,
Rocco Falchetto^4^, Edrin R. Williams^2^, Jasmin Barman-Aksözen^4,5^, Marc DeCongelio^6^,
Alison Bulkley^6^, Joana E. Matos^7^, Tarek Mnif^8^, Jordanna Mora^9^, John J. Ko^9^,
Stephen Meninger^9^, Stephen Lombardelli^10^, Danielle Nance^11^

^1^*Division of Pulmonary and Critical Care Medicine, Department of Medicine,*Massachusetts General Hospital, Boston, MA, USA

^2^American Porphyria Foundation, Bethesda, MD, USA

^3^Global Porphyria Advocacy Coalition, Durham City, UK

^4^Swiss Society for Porphyria, Zürich, Switzerland

^5^*Institute of Laboratory Medicine, Department of Medical Institutes,*
*Stadtspital Zürich, Triemli, Zürich, Switzerland*

^6^Cerner Enviza (formerly Kantar Health), New York, NY, USA

^7^Cerner Enviza (formerly Kantar Health), Kansas City, MO, USA

^8^Cerner Enviza (formerly Kantar Health), Paris, France

^9^Alnylam Pharmaceuticals, Cambridge, MA, USA

^10^Alnylam Pharmaceuticals, Maidenhead, UK

^11^Banner Health, Gilbert, AZ, USA

**CONTENTS**

**Supplementary Tables**

1 Patient demographics collected

2 Patients’ AHP-specific clinical characteristics

3 Categorical PROs

4 Continuous PROs

5 Categorical physical, emotional, and social health outcomes

6 WHYMPI scores

7 WPAI scores

8 Subgroup patient demographic and disease characteristics

9 Healthcare utilization and pain severity

**Supplementary Figures**

1 Impact on mental acuity

2 Impact on personal life/goals

3 Current health perceptions among sporadic and recurrent attack subgroups

4 Current health perceptions among prophylactic and no-prophylactic subgroups

**SUPPLEMENTARY TABLE 1** Patient demographics collected

| **Variable** | **Categories** |
| --- | --- |
| Country | United States |
|  | Mexico |
|  | Brazil |
|  | Spain |
|  | Italy |
|  | Australia |
| Gender | Male |
|  | Female |
| Employment status | Employed/homemaker/retired/student |
|  | Unemployed/disabled |
| Marital status | Married or cohabitating |
|  | Not living with partner |
| Children | Yes |
|  | No |
| Relationship to children | Biological children |
|  | Adopted children |
|  | Stepchildren |
|  | Prefer not to answer |
| Living situation | I live alone |
|  | I live with my spouse or partner |
|  | I live with my spouse or partner and children |
|  | I live with my children but without my spouse or partner |
|  | I live in my parent’s or parents’ home |
|  | I live in the home of one of my adult children |
|  | I live with someone, other than my spouse, children, or parent(s) |
|  | I live in a nursing home |
|  | Other institution |
|  | I am homeless or have insecure housing |
|  | Prefer not to answer |
| Menstrual cycles | I have menstrual cycles |
|  | I am going/have gone through menopause |
| Impact of Covid-19 | Not at all |
|  | Somewhat/very much/extremely |
| Experienced Covid-19  within past 4 weeks | Yes |
|  | No/I don’t know |

**SUPPLEMENTARY TABLE 2** Patients’ AHP-specific clinical characteristics

| **Variable** | **Category** |
| --- | --- |
| Age | — |
| Age first experienced symptoms/signs of AHP | — |
| Age at diagnosis | — |
| Time to diagnosis (raw) | — |
| Time to diagnosis (recoded to zero) | — |
| Number of patients diagnosed before experiencing symptoms | — |
| Duration of active disease | — |
| Time since first treatment | — |
| Age first received treatment | — |
| IV hemin therapy | — |
| Age first received | — |
| Amount of time receiving | — |
| IV glucose | — |
| Age first received | — |
| Amount of time receiving | — |
| AHP attacks within past 2 years | — |
| Number of attacks leading to: | — |
| Hospitalization | — |
| Emergency department visit | — |
| Doctor visit | — |
| Self-management | — |
| AHP diagnosis | Acute intermittent porphyria |
|  | Hereditary coproporphyria/variegate porphyria/5'-aminolevulinic acid dehydratase deficiency porphyria |
| AHP diagnostic tests | Urinary aminolevulinic acid test (eg, Mauzerall-Granick test) |
|  | Urinary porphobilinogen test (eg, Watson-Schwartz test, Hoesch test, Mauzerall-Granick test) |
|  | Other biochemical testing (ie, urinary porphyrins alone, stool or plasma testing) |
|  | Genetic testing performed by a healthcare professional |
|  | Other genetic testing (23andMe, AncestryDNA, FamilyTreeDNA) |
|  | A diagnostic test has been performed, but I don’t remember which |
| AHP treatments | Routine or scheduled hemin |
|  | On-demand hemin |
|  | Routine or scheduled IV glucose |
|  | On-demand IV glucose, as needed for an attack |
|  | Gonadotropin-releasing hormone agonist |
|  | Holistic therapies |
|  | Trigger avoidance |
|  | I take treatment to relieve my symptoms |
|  | Other |
| Prophylactic use | Group 1: prophylactic |
|  | Group 2: nonprophylactic |
| Age first experienced symptoms/ signs of AHP—do not know | No |
|  | Yes |
| Age at diagnosis— do not know | No |
|  | Yes |
| Age first received treatment— do not know | No |
|  | Yes |
| Age first received IV hemin— do not know | No |
|  | Yes |
| Age first received IV glucose— do not know | No |
|  | Yes |

AHP, acute hepatic porphyria; IV, intravenous.

**SUPPLEMENTARY TABLE 3** Categorical PROs

| **Variable** | **Top 3 rank** |
| --- | --- |
|  |  |
| **Acute symptoms—most burdensome** | Yes/No |
| Pain | Yes |
| Muscle weakness | Yes |
| Fatigue/tiredness | Yes |
| Vomiting | Yes |
| Emotional distress, including anxiety and/or depression | Yes |
| Profound confusion or brain dysfunction | Yes |
| Constipation | Yes |
| Nausea | Yes |
| Paralysis | Yes |
| Fast heartbeat | Yes |
| Headache | Yes |
| Convulsions or seizures | Yes |
| Trouble sleeping | Yes |
| Hallucinations | Yes |
| Diarrhea | Yes |
| Sweating | Yes |
| Blisters or rashes | Yes |
| Sun sensitivity | Yes |
| Other | Yes |
| **Chronic symptoms—most burdensome** |  |
| Pain | Yes |
| Fatigue/tiredness | Yes |
| Muscle weakness | Yes |
| Emotional distress, including anxiety and/or depression | Yes |
| Headache | Yes |
| Trouble sleeping | Yes |
| Constipation | Yes |
| Paralysis | Yes |
| Sweating | Yes |
| Nausea | Yes |
| Profound confusion or brain dysfunction | Yes |
| Vomiting | Yes |
| Fast heartbeat | Yes |
| Sun sensitivity | Yes |
| Convulsions or seizures | Yes |
| Diarrhea | Yes |
| Blisters or rashes | Yes |
| Other | Yes |
| **Chronic symptom severity (1 vs 2–5)—analysis** |  |
| Pain | No symptom |
|  | Symptom |
| Muscle weakness | No symptom |
|  | Symptom |
| Paralysis | No symptom |
|  | Symptom |
| Fatigue/tiredness | No symptom |
|  | Symptom |
| Nausea | No symptom |
|  | Symptom |
| Vomiting | No symptom |
|  | Symptom |
| Profound confusion or brain dysfunction | No symptom |
|  | Symptom |
| Convulsions or seizures | No symptom |
|  | Symptom |
| Trouble sleeping | No symptom |
|  | Symptom |
| Emotional distress, including anxiety and/or depression | No symptom |
|  | Symptom |
| Headache | No symptom |
|  | Symptom |
| Constipation | No symptom |
|  | Symptom |
| Diarrhea | No symptom |
|  | Symptom |
| Fast heartbeat | No symptom |
|  | Symptom |
| Sweating | No symptom |
|  | Symptom |
| Blisters or rashes | No symptom |
|  | Symptom |
| Sun sensitivity | No symptom |
|  | Symptom |
| Other | No symptom |
|  | Symptom |
| **Chronic symptom severity (1 vs 3–5)—analysis** |  |
| Pain | No limit on activities |
|  | Limit on activities |
| Muscle weakness | No limit on activities |
|  | Limit on activities |
| Paralysis | No limit on activities |
|  | Limit on activities |
| Fatigue/tiredness | No limit on activities |
|  | Limit on activities |
| Nausea | No limit on activities |
|  | Limit on activities |
| Vomiting | No limit on activities |
|  | Limit on activities |
| Profound confusion or brain dysfunction | No limit on activities |
|  | Limit on activities |
| Convulsions or seizures | No limit on activities |
|  | Limit on activities |
| Trouble sleeping | No limit on activities |
|  | Limit on activities |
| Emotional distress, including anxiety and/or depression | No limit on activities |
|  | Limit on activities |
| Headache | No limit on activities |
|  | Limit on activities |
| Constipation | No limit on activities |
|  | Limit on activities |
| Diarrhea | No limit on activities |
|  | Limit on activities |
| Fast heartbeat | No limit on activities |
|  | Limit on activities |
| Sweating | No limit on activities |
|  | Limit on activities |
| Blisters or rashes | No limit on activities |
|  | Limit on activities |
| Sun sensitivity | No limit on activities |
|  | Limit on activities |
| Other | No limit on activities |
|  | Limit on activities |
| **Chronic symptom severity (3 vs 4 vs 5)—analysis** |  |
| Pain | Mild |
|  | Moderate |
|  | Severe |
| Muscle weakness | Mild |
|  | Moderate |
|  | Severe |
| Paralysis | Mild |
|  | Moderate |
|  | Severe |
| Fatigue/tiredness | Mild |
|  | Moderate |
|  | Severe |
| Nausea | Mild |
|  | Moderate |
|  | Severe |
| Vomiting | Mild |
|  | Moderate |
|  | Severe |
| Profound confusion or brain dysfunction | Mild |
|  | Moderate |
|  | Severe |
| Convulsions or seizures | Mild |
|  | Moderate |
|  | Severe |
| Trouble sleeping | Mild |
|  | Moderate |
|  | Severe |
| Emotional distress, including anxiety and/or depression | Mild |
|  | Moderate |
|  | Severe |
| Headache | Mild |
|  | Moderate |
|  | Severe |
| Constipation | Mild |
|  | Moderate |
|  | Severe |
| Diarrhea | Mild |
|  | Moderate |
|  | Severe |

| Fast heartbeat | Mild |
| --- | --- |
|  | Moderate |
|  | Severe |
| Sweating | Mild |
|  | Moderate |
|  | Severe |
| Blisters or rashes | Mild |
|  | Moderate |
|  | Severe |
| Sun sensitivity | Mild |
|  | Moderate |
|  | Severe |
| Other | Mild |
|  | Moderate |
|  | Severe |
| **Health perceptions—current health** |  |
| Physical health | Poor, fair |
|  | Good, very good, excellent |
| Emotional health | Poor, fair |
|  | Good, very good, excellent |
| Cognitive health | Poor, fair |
|  | Good, very good, excellent |
| Financial health | Poor, fair |
|  | Good, very good, excellent |
| Social health | Poor, fair |
|  | Good, very good, excellent |
| PHQ-8 (8-item Patient Health Questionnaire depression scale) | <10 |
|  | ≥10 |
| GAD-7 (7-item Generalized Anxiety Disorder scale) | 0–4 (minimal anxiety) |
|  | 5–9 (mild anxiety) |
|  | 10–14 (moderate anxiety) |
|  | 15+ (severe anxiety) |

PRO, patient-reported outcome.

**SUPPLEMENTARY TABLE 4** Continuous PROs

| **Variable** |
| --- |
| Depression and anxiety screeners |
| PHQ-8, continuous |
| GAD-7, continuous |
| Pain scale: WHYMPI |
| Interference |
| Support |
| Pain severity |
| Life-control |
| Affective distress |
| WPAI |
| Absenteeism |
| Presenteeism |
| Overall work productivity impairment |
| Activity impairment |

GAD-7, 7-item Generalized Anxiety Disorder scale; PHQ-8, 8-item Patient Health Questionnaire depression scale; PRO, patient-reported outcome; WHYMPI, West Haven–Yale Multidimensional Pain Inventory; WPAI, Work Productivity and Activity Impairment.

**SUPPLEMENTARY TABLE 5** Categorical physical, emotional, and social health outcomes

| **Social life** | |
| --- | --- |
| I appreciate it when my friends and family bring me new information about how to manage my AHP | *Strongly disagree/disagree*  *Neither agree nor disagree*  *Strongly agree/agree* |
| I feel like my friends and family give me the support that I need | *Strongly disagree/disagree*  *Neither agree nor disagree*  *Strongly agree/agree* |
| I often hide or avoid discussing my AHP with people close to me | *Strongly disagree/disagree*  *Neither agree nor disagree*  *Strongly agree/agree* |
| I am able to make new friends despite having AHP | *Strongly disagree/disagree*  *Neither agree nor disagree*  *Strongly agree/agree* |
| When I tell my friends and family about the challenges of my AHP,  I feel like they think I am complaining | *Strongly disagree/disagree*  *Neither agree nor disagree*  *Strongly agree/agree* |
| I feel frustrated that people close to me (e.g., friends, employer) do not understand the challenges I face because most of my AHP symptoms are not visible | *Strongly disagree/disagree*  *Neither agree nor disagree*  *Strongly agree/agree* |
| I often feel guilty and upset for how my symptoms and disabilities affect others around me | *Strongly disagree/disagree*  *Neither agree nor disagree*  *Strongly agree/agree* |
| I often feel lonely or isolated | *Strongly disagree/disagree*  *Neither agree nor disagree*  *Strongly agree/agree* |
| I believe my AHP has caused my family members to have anxiety or depression | *Strongly disagree/disagree*  *Neither agree nor disagree*  *Strongly agree/agree* |
| I go out of my way to keep my AHP private and hidden from most people in my day-to-day life | *Strongly disagree/disagree*  *Neither agree nor disagree*  *Strongly agree/agree* |
| Most of my AHP symptoms are hidden and most people do not even know that I have AHP | *Strongly disagree/disagree*  *Neither agree nor disagree*  *Strongly agree/agree* |
| **Employment/career** | |
| My AHP has affected my ability to keep my job | *Strongly disagree/disagree*  *Neither agree nor disagree*  *Strongly agree/agree* |
| I cannot handle all the work I am assigned or expected to complete at my job or volunteer position due to my AHP | *Strongly disagree/disagree*  *Neither agree nor disagree*  *Strongly agree/agree* |
| I had to change jobs due to my AHP | *Strongly disagree/disagree*  *Neither agree nor disagree*  *Strongly agree/agree* |
| I am not concerned about being able to keep my job/volunteer position | *Strongly disagree/disagree*  *Neither agree nor disagree*  *Strongly agree/agree* |
| I feel pride in my job or career/volunteer position | *Strongly disagree/disagree*  *Neither agree nor disagree*  *Strongly agree/agree* |
| I actively seek to progress my career, regardless of my AHP | *Strongly disagree/disagree*  *Neither agree nor disagree*  *Strongly agree/agree* |
| I am seeking employment | *Strongly disagree/disagree*  *Neither agree nor disagree*  *Strongly agree/agree* |
| I feel I am not able to work to my full potential because of my AHP | *Strongly disagree/disagree*  *Neither agree nor disagree*  *Strongly agree/agree* |
| I am afraid I could lose my job/volunteer position or not get promoted if my employer finds out about my AHP | *Strongly disagree/disagree*  *Neither agree nor disagree*  *Strongly agree/agree* |
| **Fatigue** | |
| I am easily overwhelmed by everyday tasks | *Strongly disagree/disagree*  *Neither agree nor disagree*  *Strongly agree/agree* |
| I am unable to do housework or chores because of my fatigue | *Strongly disagree/disagree*  *Neither agree nor disagree*  *Strongly agree/agree* |
| I have enough energy to do what I need to during the day | *Strongly disagree/disagree*  *Neither agree nor disagree*  *Strongly agree/agree* |
| I often take naps during the day | *Strongly disagree/disagree*  *Neither agree nor disagree*  *Strongly agree/agree* |
| **Sleep** | |
| It is difficult for me to stay asleep | *Strongly disagree/disagree*  *Neither agree nor disagree*  *Strongly agree/agree* |
| I put off going to bed at night for fear of not being able to sleep | *Strongly disagree/disagree*  *Neither agree nor disagree*  *Strongly agree/agree* |
| My inability to sleep interferes with daily functioning | *Strongly disagree/disagree*  *Neither agree nor disagree*  *Strongly agree/agree* |
| I don’t sleep due to physical symptoms | *Strongly disagree/disagree*  *Neither agree nor disagree*  *Strongly agree/agree* |
| I don’t sleep due to emotional symptoms/worry about my AHP | *Strongly disagree/disagree*  *Neither agree nor disagree*  *Strongly agree/agree* |
| **Personal life/goals** | |
| I am committed to a healthy diet and exercise choices to help prevent AHP symptoms | *Strongly disagree/disagree*  *Neither agree nor disagree*  *Strongly agree/agree* |
| I spend more time focusing on what DOES work with my body or mind than what DOES NOT | *Strongly disagree/disagree*  *Neither agree nor disagree*  *Strongly agree/agree* |
| I have trouble keeping a schedule due to my AHP | *Strongly disagree/disagree*  *Neither agree nor disagree*  *Strongly agree/agree* |
| The decline in my mental and physical health feels never ending | *Strongly disagree/disagree*  *Neither agree nor disagree*  *Strongly agree/agree* |
| AHP has pushed me to find a new sense of purpose | *Strongly disagree/disagree*  *Neither agree nor disagree*  *Strongly agree/agree* |
| I have lost my sense of purpose due to AHP | *Strongly disagree/disagree*  *Neither agree nor disagree*  *Strongly agree/agree* |
| I have had to change or modify goals that are important to me because of my AHP | *Strongly disagree/disagree*  *Neither agree nor disagree*  *Strongly agree/agree* |
| **Mental acuity** | |
| I am able to maintain focus on projects or tasks for the period of time required to complete them | *Strongly disagree/disagree*  *Neither agree nor disagree*  *Strongly agree/agree* |
| I do not have difficulties concentrating and remembering things | *Strongly disagree/disagree*  *Neither agree nor disagree*  *Strongly agree/agree* |
| My ability to process information often leaves me feeling like I cannot keep up with social activities (e.g., participating in group discussion, “getting the joke” as quickly as others do) | *Strongly disagree/disagree*  *Neither agree nor disagree*  *Strongly agree/agree* |
| I sometimes have trouble making decisions | *Strongly disagree/disagree*  *Neither agree nor disagree*  *Strongly agree/agree* |
| I sometimes forget names or words for common objects | *Strongly disagree/disagree*  *Neither agree nor disagree*  *Strongly agree/agree* |
| **Financial well-being** | |
| Having AHP has not caused me financial difficulties | *Strongly disagree/disagree*  *Neither agree nor disagree*  *Strongly agree/agree* |
| My AHP has left me with medical debt | *Strongly disagree/disagree*  *Neither agree nor disagree*  *Strongly agree/agree* |
| I have had to rely on other people financially due to my AHP | *Strongly disagree/disagree*  *Neither agree nor disagree*  *Strongly agree/agree* |
| Due to my AHP, I rely on government assistance | *Strongly disagree/disagree*  *Neither agree nor disagree*  *Strongly agree/agree* |
| **Mobility** | |
| My AHP has not affected how mobile I am | *Strongly disagree/disagree*  *Neither agree nor disagree*  *Strongly agree/agree* |
| I have had to use a cane or wheelchair due to AHP | *Strongly disagree/disagree*  *Neither agree nor disagree*  *Strongly agree/agree* |
| **School (for students only)** | |
| My AHP has affected my ability to go to school | *Strongly disagree/disagree*  *Neither agree nor disagree*  *Strongly agree/agree* |
| I cannot handle all the work I am assigned or expected to complete at school due to my AHP | *Strongly disagree/disagree*  *Neither agree nor disagree*  *Strongly agree/agree* |
| I am not concerned about being able to keep going to school | *Strongly disagree/disagree*  *Neither agree nor disagree*  *Strongly agree/agree* |

**SUPPLEMENTARY TABLE 6** WHYMPI scores

| **Subscale** | **Overall** | | **Attack rate** | | | | **Prophylactic treatment** | | | |
| --- | --- | --- | --- | --- | --- | --- | --- | --- | --- | --- |
|  |  |  | **Sporadic** | | **Recurrent** | | **Prophylaxis** | | **No prophylaxis** | |
|  | **Mean** | **SD** | **Mean** | **SD** | **Mean** | **SD** | **Mean** | **SD** | **Mean** | **SD** |
| Interference | 3.62 | 1.75 | 3.22 | 1.81 | 4.22 | 1.48 | 4.04 | 1.27 | 3.36 | 1.95 |
| Support | 4.45 | 1.63 | 4.40 | 1.63 | 4.52 | 1.64 | 4.53 | 1.82 | 4.40 | 1.51 |
| Pain severity | 3.43 | 1.63 | 2.93 | 1.68 | 4.17 | 1.25 | 3.84 | 1.33 | 3.18 | 1.76 |
| Life-control | 3.30 | 1.71 | 3.64 | 1.77 | 2.81 | 1.51 | 3.19 | 1.57 | 3.38 | 1.80 |
| Affective distress | 3.66 | 0.80 | 3.55 | 0.88 | 3.82 | 0.65 | 3.80 | 0.65 | 3.57 | 0.88 |

SD, standard deviation; WHYMPI, West Haven–Yale Multidimensional Pain Inventory.

**SUPPLEMENTARY TABLE 7** WPAI scores

| **Domain** | **Overall** | | **Attack rate** | | | | **Prophylactic treatment** | | | |
| --- | --- | --- | --- | --- | --- | --- | --- | --- | --- | --- |
|  |  |  | **Sporadic** | | **Recurrent** | | **Prophylaxis** | | **No prophylaxis** | |
|  | **N** | **Mean % (SD)** | **N** | **Mean % (SD)** | **N** | **Mean % (SD)** | **N** | **Mean % (SD)** | **N** | **Mean % (SD)** |
| Absenteeism | 43 | 32.6 (35.9) | 26 | 37.5 (39.2) | 17 | 25.1 (29.6) | 14 | 34.4 (37.1) | 29 | 31.8 (35.9) |
| Presenteeism | 38 | 36.8 (34.1) | 21 | 30.0 (31.8) | 17 | 45.3 (35.9) | 13 | 46.2 (36.2) | 25 | 32.0 (32.7) |
| Overall work productivity impairment | 43 | 52.3 (39.1) | 26 | 51.5 (40.9) | 17 | 53.5 (37.2) | 14 | 61.6 (35.2) | 29 | 47.8 (40.6) |
| Activity impairment | 81 | 51.6 (35.3) | 46 | 44.1 (35.1) | 35 | 61.4 (33.5) | 32 | 57.5 (32.6) | 49 | 47.8 (36.7) |

SD, standard deviation; WPAI, Work Productivity and Activity Impairment.

**SUPPLEMENTARY TABLE 8** Subgroup patient demographic and disease characteristics

| **Characteristic** | **Attack rate** | | **Prophylactic treatment** | |
| --- | --- | --- | --- | --- |
|  | **0–5/2 years**  **N=55** | **≥6/2 years**  **N=37** | **Prophylaxis**  **N=35** | **No prophylaxis**  **N=57** |
| Age, mean (SD), years | 40.3 (12.3) | 42.3 (12.5) | 41.0 (11.8) | 41.1 (12.8) |
| Female, n (%) | 51 (92.7) | 32 (86.5) | 31 (88.6) | 52 (91.2) |
| Diagnosis, n (%) |  |  |  |  |
| Acute intermittent porphyria | 46 (85.2) | 22 (61.1) | 20 (58.8) | 48 (85.7) |
| Hereditary coproporphyria  Variegate porphyria  5'-Aminolevulinic acid dehydratase deficiency porphyria | 8 (14.8) | 14 (38.9) | 14 (41.2) | 8 (14.3) |
| Age at diagnosis, mean (SD), years | 30.2 (9.9) | 31.8 (12.2) | 29.5 (11.3) | 31.6 (10.6) |
| Time to diagnosis, mean (SD), years | 5.2 (10.3) | 8.0 (9.8) | 6.1 (8.9) | 6.5 (10.9) |
| Duration of disease, mean (SD), years | 15.7 (12.6) | 18.4 (13.6) | 17.7 (11.9) | 16.3 (13.8) |

SD, standard deviation.

**SUPPLEMENTARY TABLE 9** Healthcare utilization and pain severity

| **Variable** | **Attack rate** | | | | | | | | ***P*-value attack rate** |
| --- | --- | --- | --- | --- | --- | --- | --- | --- | --- |
|  | **0–5/2 years N=55** | | | | **≥6/2 years N=37** | | | |  |
|  | **N** | **Mean** | **SD** | **Median** | **N** | **Mean** | **SD** | **Median** |  |
| **Number of attacks leading to:** |  |  |  |  |  |  |  |  |  |
| Hospitalization | 49 | 1.63 | 1.45 | 1.00 | 37 | 4.00 | 4.50 | 3.00 | 0.001 |
| Emergency department visit | 49 | 1.61 | 1.51 | 1.00 | 37 | 6.41 | 7.60 | 4.00 | 0.000 |
| **WHYMPI pain severity** | 55 | 2.93 | 1.68 | 3.00 | 37 | 4.17 | 1.25 | 4.33 | 0.000 |

AHP, acute hepatic porphyria; SD, standard deviation; WHYMPI, West Haven–Yale Multidimensional Pain Inventory.

**SUPPLEMENTARY FIGURE 1** Impact on mental acuity.

**SUPPLEMENTARY FIGURE 2** Impact on personal life/goals.

**SUPPLEMENTARY FIGURE 3** Current health perceptions among sporadic and recurrent attack subgroups. Each health aspect was categorized as poor, fair, good, very good, or excellent. Data presented represent percentages of patients who reported fair or poor perception of their health. Complementary values for each category represent good, very good, or excellent perception of health.

**SUPPLEMENTARY FIGURE 4** Current health perceptions among prophylactic and no-prophylactic subgroups. Each health aspect was categorized as poor, fair, good, very good, or excellent. Data presented represent percentages of patients who reported fair or poor perception of their health. Complementary values for each category represent good, very good, or excellent perception of health.
